# Supplementary material for: Transcriptional activator TAp63 is upregulated in muscular atrophy during ALS and induces the pro-atrophic ubiquitin ligase Trim63
Source: eLife. 2016 Feb 26;5:e10528. doi: 10.7554/eLife.10528 (PMC4786414; doi:10.7554/eLife.10528)
Supplement: Supplementary file 1. — DOI: http://dx.doi.org/10.7554/eLife.10528.021 [file elife-10528-supp1.docx]

| ***qPCR primers*** | **forward** | **reverse** |
| --- | --- | --- |
| *18S* | cgtctgccctatcaactttcg | ttccttggatgtggtagccg |
| *Tbp* | ccaatgactcctatgaccccta | cagccaagattcacggtagat |
| *Chrna1* | ccacagactcaggggagaag | aacggtggtgtgtgttgatg |
| *Fbxo32* (Atrogin1) | agtgaggaccggctactgtg | gatcaaacgcttgcgaatct |
| *Bax* | atgcgtccaccaagaagctga | agcaatcatcctctgcagctcc |
| *Bip/Grp78* | ctgaggcgtatttgggaaag | tcatgacattcagtccagcaa |
| *Chop/ddit3/gadd153* | gcgacagagccagaataaca | gatgcacttccttctggaaca |
| *Dr5* | cgggcagatcactacaccc | gttactggaacaaagacagcc |
| *Eda2R* | ctgtgtcacctgccaacaa | gggagggcagactatgcag |
| *GADD45a* | gctgccaagctgctcaac | tcgtcgtcttcgtcagca |
| *Id2* | gacagaaccaggcgtcca | agctcagaagggaattcagatg |
| *Mdm2* | ttagtggctgtaagtcagcaaga | tcagatcactcccaccttca |
| *Mlf1* | ccgcaggatcacgatgtt | tctcggtgtgcaagaaaaga |
| *Trim63* (Murf1) | gcaggagtgctccagtcg | tcttcgtgttccttgcacat |
| *Myf6* | gggcctcgtgataactgct | aagaaaggcgctgaagactg |
| *Pmaip1* | ccacctgagttcgcagctcaa | gttgagcacactcgtccttcaa |
| *Cdkn1a* | tccacagcgatatccagaca | ggacatcaccaggattggac |
| *p53* | acgcttctccgaagactgg | agggagctcgaggctgata |
| TAp63 | actttgtggatgaaccttccg | ctgtgcgtggtctgtgttgtag |
| ΔNp63 | tgagccacagtacacgaacc | gagagagggcatcaaaggtg |
| TAp73 | cccagccaagggactagc | gcactgctgagcaaattgaa |
| ΔNp73 | agatgggcagggtgagaatg | cagacagcacctactttgacctc |
| *Peg3* | gcctcagaatccaattcagg | tcttcggcaagctggact |
| *Puma* | agcagcacttagagtcgcc | cctgggtaaggggaggagt |
| *Sco2* | atcgcacagccctaagtctc | cagtagcatcgtggacctga |
| *Sestrin1* | tgtcccaacgtttcgtgtc | tggatagagacgattcaccaga |
| *Sestrin2* | acatccactgcgtctttgg | cgtcttgatatagattttgaggttcc |
| *Siva* | caggagtgcttgcagggta | gcatgaatcagccctaaggt |
| *Tigar* | tgtaaagacatggcggtgaa | cgcgactccgtacatcct |
| *Txn1* | tgaagctgatcgagagcaag | agaagtccaccacgacaagc |
| *xbp1* | tgacgaggttccagaggtg | tgcagaggtgcacatagtctg |
| *Zmat3/Wig1* | cacacccctcgttcctgt | ttctctgtagccaggatcacc |
|  |  |  |
| ***ChIP assay*** |  |  |
| *p63-RE1+2* | cagcacaagggtgttcatgt | tcagtggtaaaggggcttgct |
| *p63-RE4* | cctttgtttcaaagacctccttc | ggccagtctcaggaaaagg |

Primers for qPCR
